# Supplementary material for: Rate and Product Studies with 1-Adamantyl Chlorothioformate under Solvolytic Conditions
Source: Int J Mol Sci. 2021 Jul 9;22(14):7394. doi: 10.3390/ijms22147394 (PMC8306509; doi:10.3390/ijms22147394)
Supplement: Supplementary file 1 [file ijms-22-07394-s001.zip › ijms-1261472-SI.pdf]

Supporting Materials

Table S1. Calculated energy, charge and distance for 1-adamantyl chlorothioformate (**1**) and chloroformate (**2**), as calculated at B3LYP/6-31G(d) level theory

**Sub.**[a]	**Energy**[b]	**Charge**[c]					**Distance (Å)**			
**1**	-361.838028	**(R)C*-S**	**S*-C=O**	**C*=O**	**C=O***	**C-X***	**(R)C-S**	**(S-C)=O**	**(C=O)**	**C-X**
		-0.130	0.199	0.143	-0.336	-0.06	1.880	1.771	1.19106	1.828
**2**	-038.874069	**(R)C*-O**	**O*-C=O**	**C*=O**	**C=O***	**C-X***	**(R)C-O**	**(O-C)=O**	**(C=O)**	**C-X**
		0.303	-0.451	0.478	-0.372	-0.083	1.484	1.3176	1.19149	1.82654

[a] 1-Adamantyl chlorothioforamte and 1-adamantyl chloroformate in the gas phase. [b] hartree (HF) = 627.5095 kcal/mol. [c]Mulliken charge.

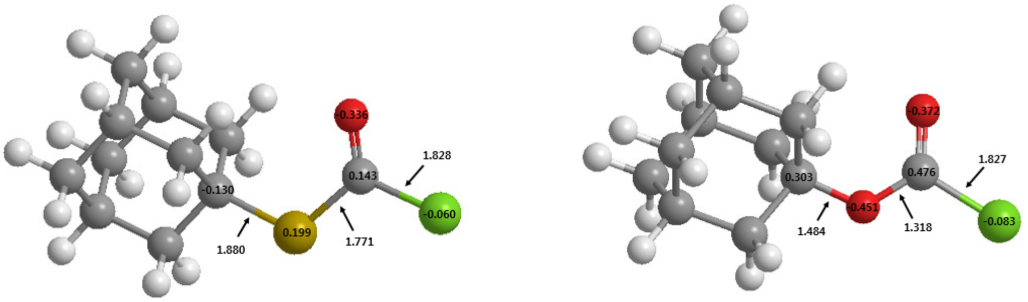

(a) (b)

Figure S2. Optimized molecular structures of (a) 1-AdSCOCl and (b) 1-AdOCOCl using B3LYP/6-31G(d).

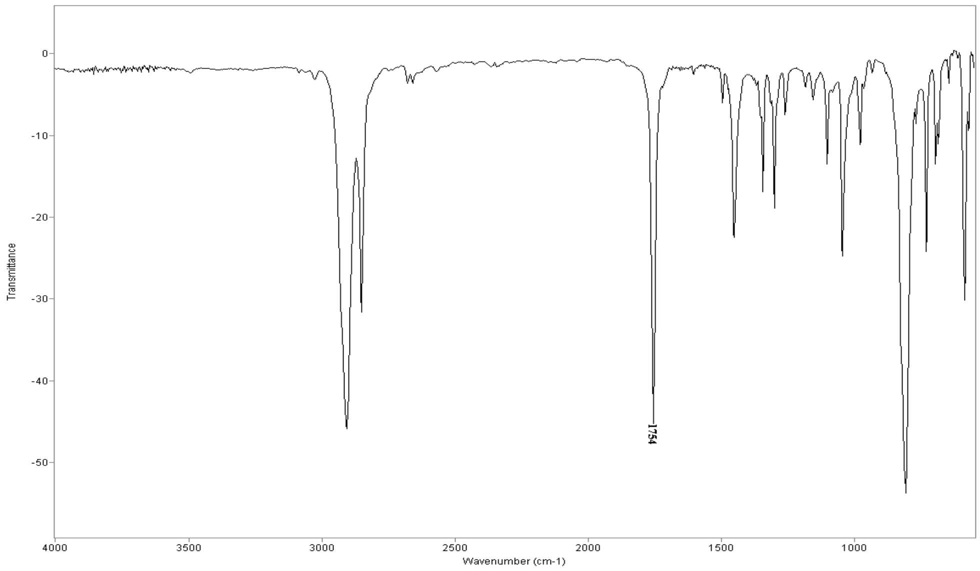

Figure S3. IR spectrum of 1-adamantyl chlorothioformate showing characteristic C=O peak at 1754 cm^{-1}.

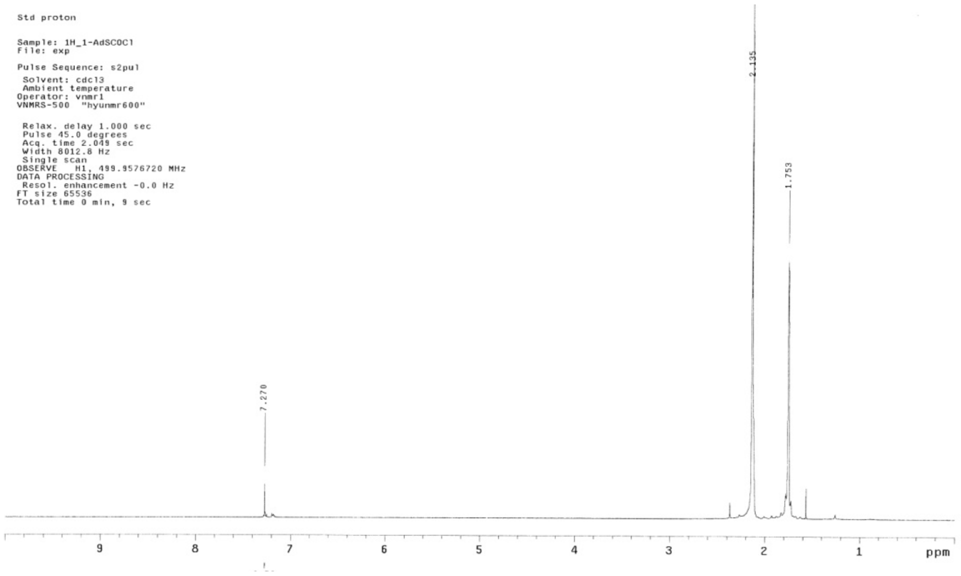

Figure S4. ^{1}H NMR spectrum of 1-adamantyl chlorothioformate.

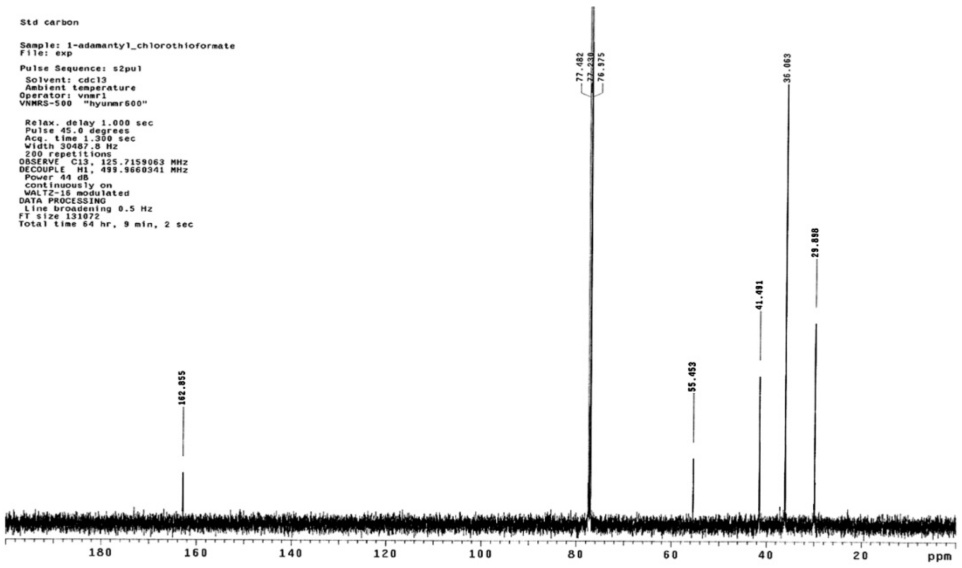

Figure S5. ^{13}C NMR spectrum of 1-adamantyl chlorothioformate.

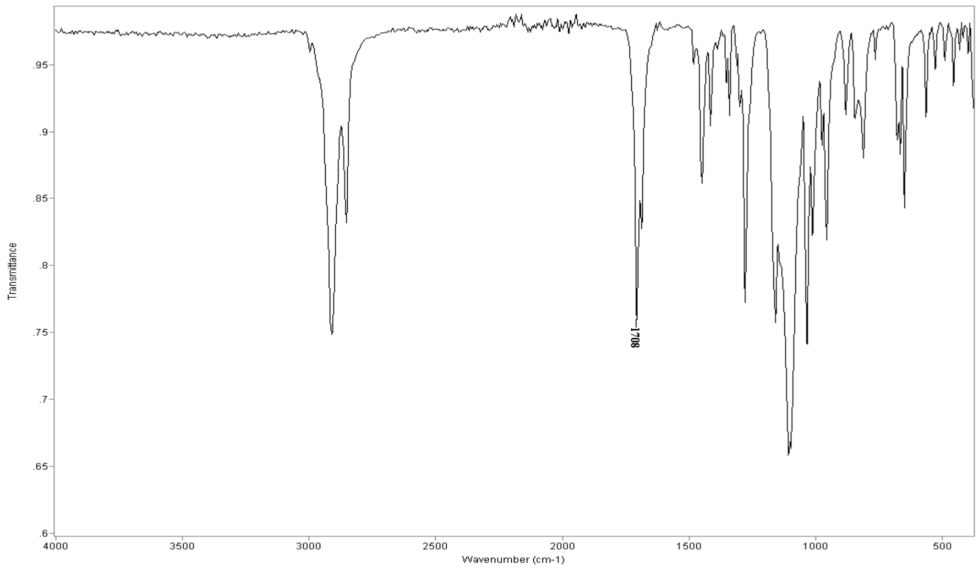

Figure S6. IR spectrum of 1-adamantyl ethyl thiocarbonate showing characteristic C=O peak at 1708 cm^{-1}.

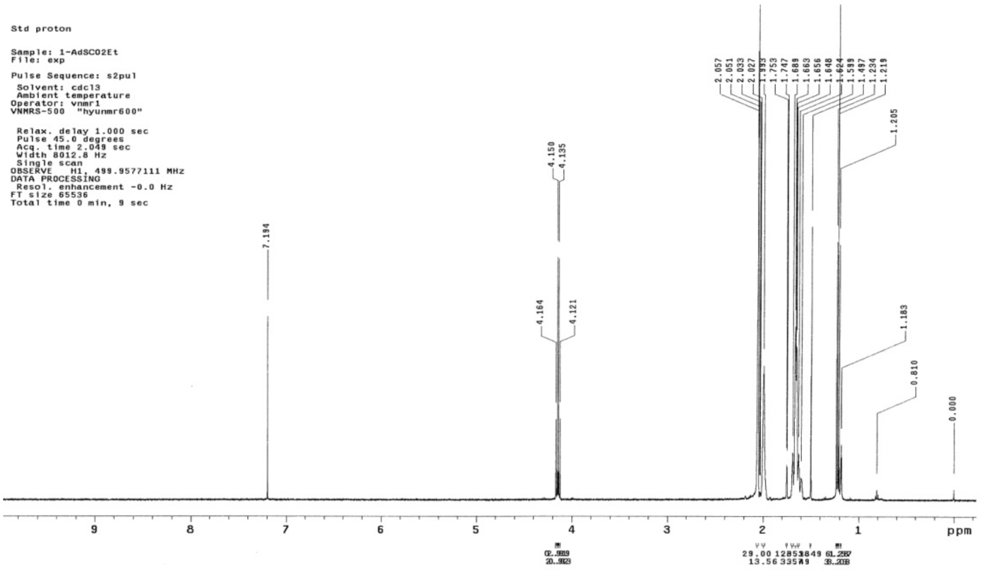

Figure S7. ^{1}H NMR spectrum of 1-adamantyl ethyl thiocarbonate.

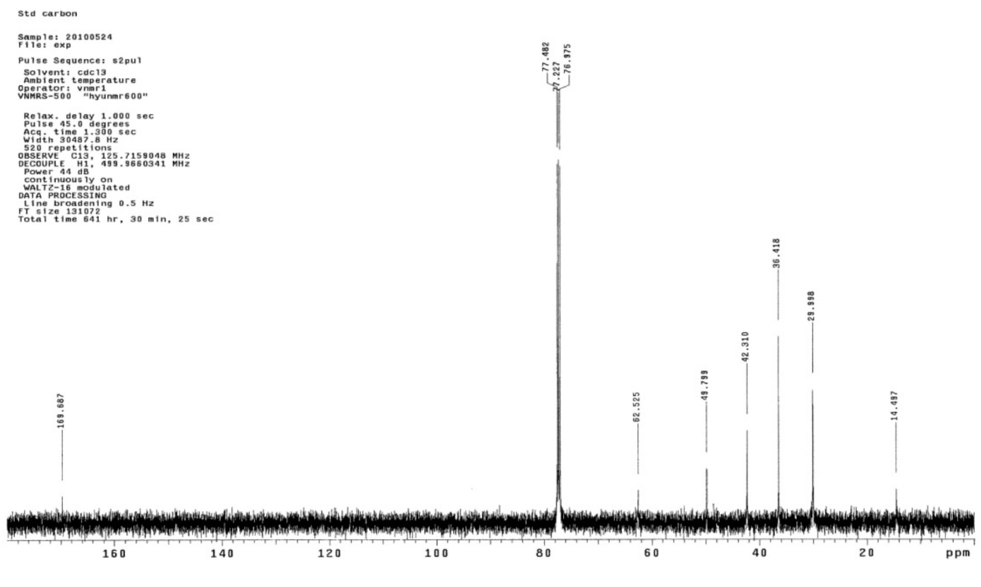

Figure S8. ^{13}C NMR spectrum of 1-adamantyl ethyl thiocarbonate.

**Substrate:** 1-adamantyl ethyl thiocarbonate (5.699×10^{-3}M)<br>**Solvent:** *n*-Pentane<br>**Injection Temp. :** 210 °C<br>**Column Temp. :** 170 °C<br>**Injection volume :** 1μL	**Gas chromatography :** GC-9A (Shimadzu)<br>**Column :** glass<br>(length: 2.1m, O.D: 5mm, I.D: 3mm)<br>**Stationary phase :** Carbowax 20M 10%<br>Chromosorb WAW 80/100<br>**Detector :** FID, **Carrier gas:** N_2

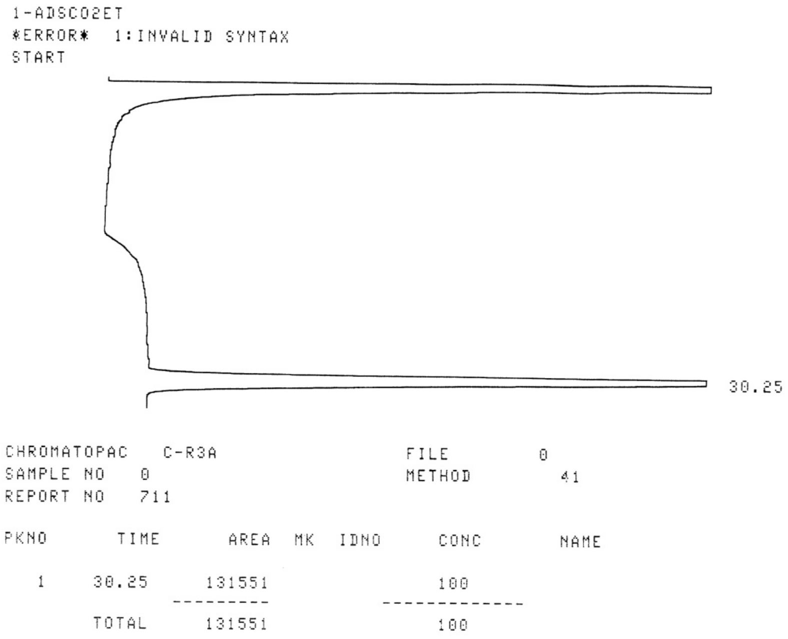

Figure S9. G.C. analysis sheet of 1-adamantyl ethyl thiocarbonate.

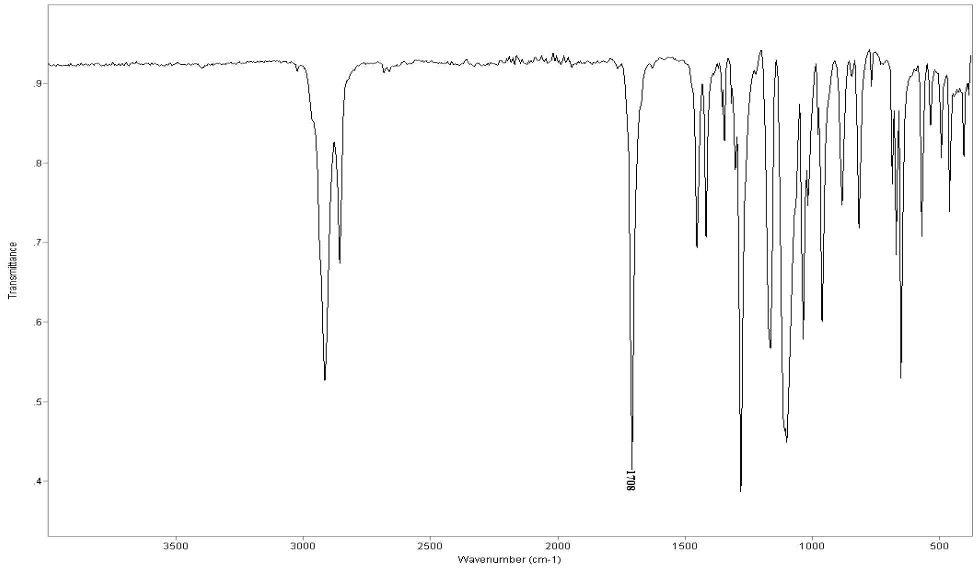

Figure S10. IR spectrum of 1-adamantyl 2,2,2-trifluoroethyl thiocarbonate showing characteristic C=O peak at 1708 cm^{-1}.

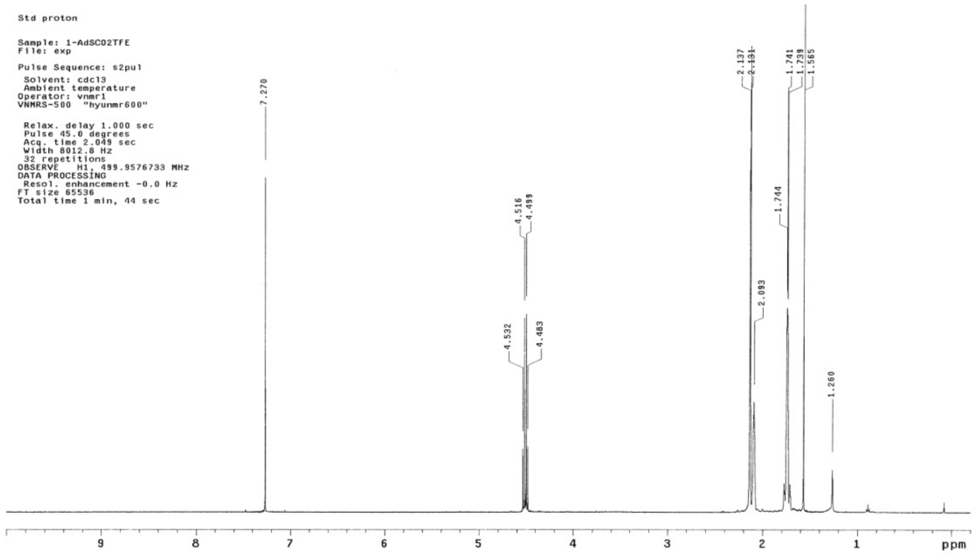

Figure S11. ^{1}H NMR spectrum of 1-adamantyl 2,2,2-trifluoroethyl thiocarbonate.

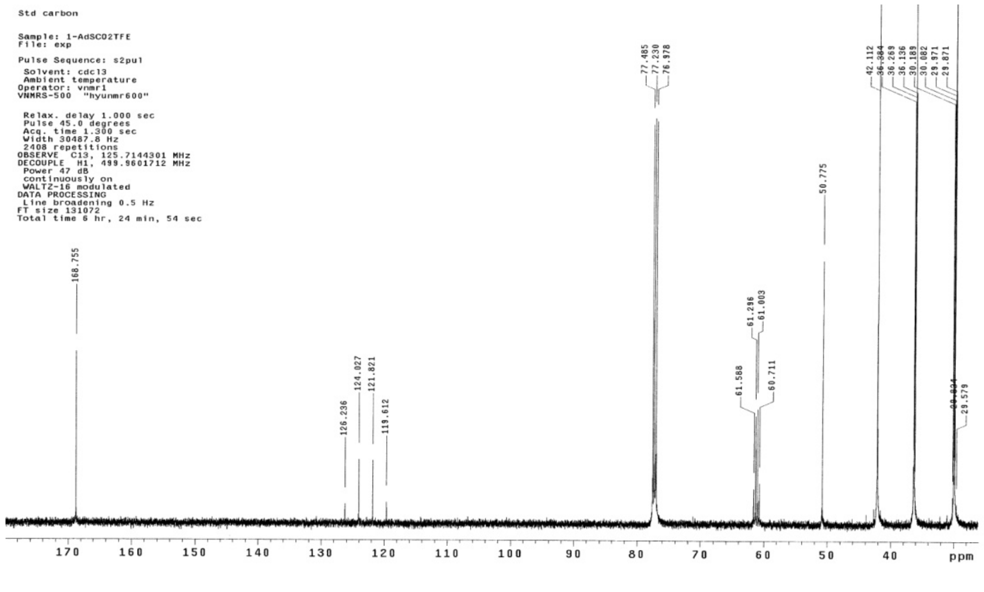

Figure S12. ^{13}C NMR spectrum of 1-adamantyl 2,2,2-trifluoroethyl thiocarbonate.

**Substrate:** 1-adamantyl 2,2,2-trifluoroethyl thiocarbonate (5.436×10^{-3}M)	**Gas chromatography :** GC-9A (Shimadzu)
**Solvent:** *n*-Pentane	**Column :** glass (length: 2.1m, O.D: 5mm, I.D: 3mm)
**Injection Temp. :** 210 °C	**Stationary phase :** Carbowax 20M 10% Chromosorb WAW 80/100
**Column Temp. :** 170 °C	
**Injection volume :** 1μL	**Detector :** FID, **Carrier gas:** N_2

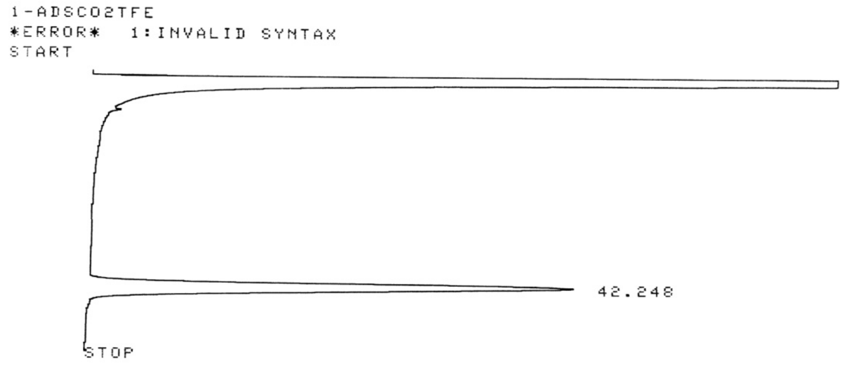

CHROMATOPAC C-R3A FILE 0
SAMPLE NO 0 METHOD 41
REPORT NO 683

PKNO	TIME	AREA	MK	IDNO	CONC	NAME
1	42.248	76001			100	
	TOTAL	76001			100	

Figure S13. G.C. analysis sheet of 1-adamantyl 2,2,2-trifluoroethyl thiocarbonate.

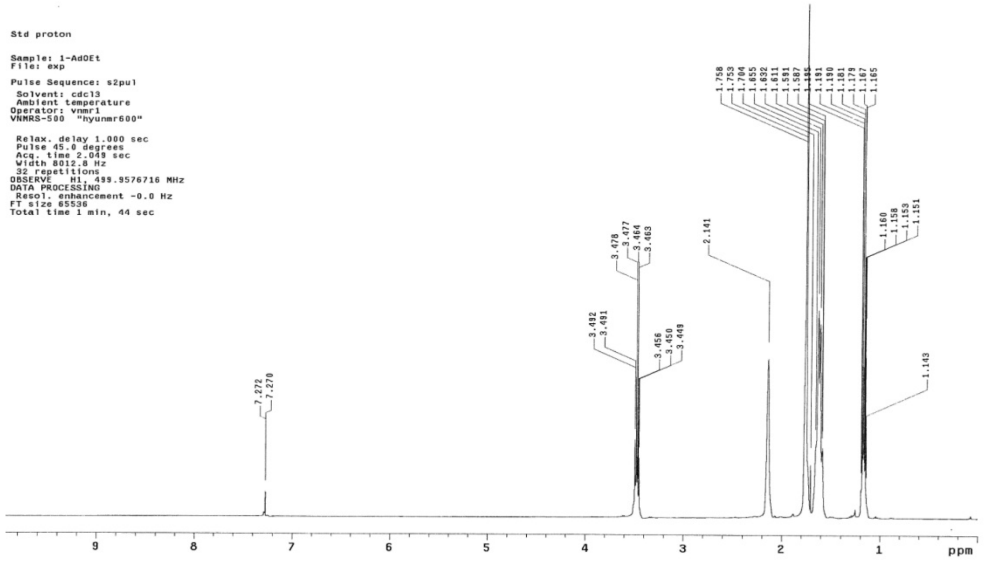

Figure S14. ^{1}H NMR spectrum of 1-adamantyl ethyl ether.

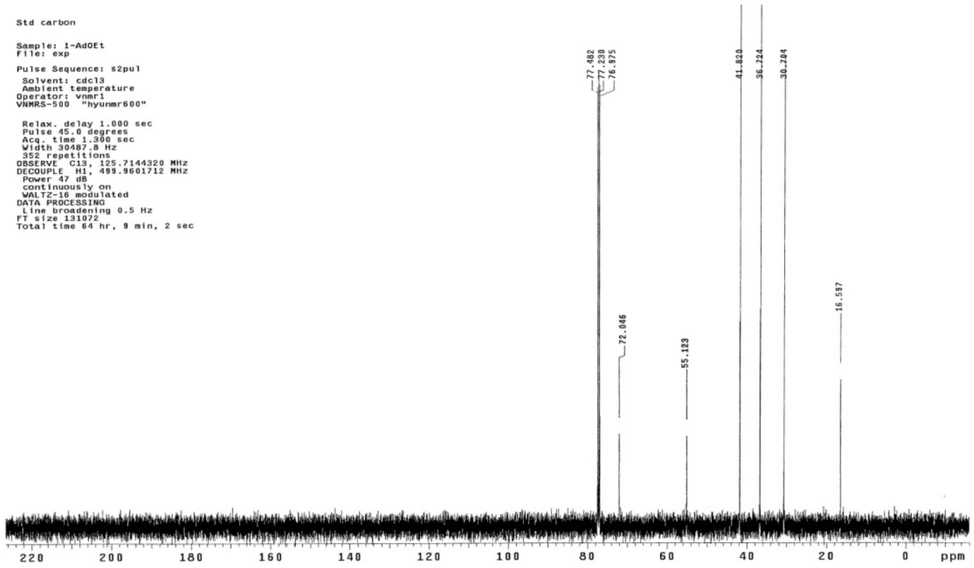

Figure S15. ^{13}C NMR spectrum of 1-adamantyl ethyl ether.

**Substrate:** 1-adamantyl ethyl ether<br>(5.547 × 10^{-3}M)<br>**Solvent:** *n*-Pentane<br>**Injection Temp. :** 210 °C<br>**Column Temp. :** 170 °C<br>**Injection volume :** 1μL	**Gas chromatography :** GC-9A (Shimadzu)<br>**Column :** glass<br>(length: 2.1m, O.D: 5mm, I.D: 3mm)<br>**Stationary phase :** Carbowax 20M 10%<br>Chromosorb WAW 80/100<br>**Detector :** FID, **Carrier gas:** N_2

START

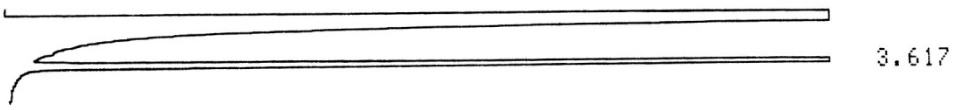

CHROMATOPAC C-R3A FILE 0
SAMPLE NO 0 METHOD 41
REPORT NO 712

PKNO	TIME	AREA	MK	IDNO	CONC	NAME
1	3.617	195621			100	
	TOTAL	195621			100	

Figure S16. G.C. analysis sheet of 1-adamantyl ethyl ether.

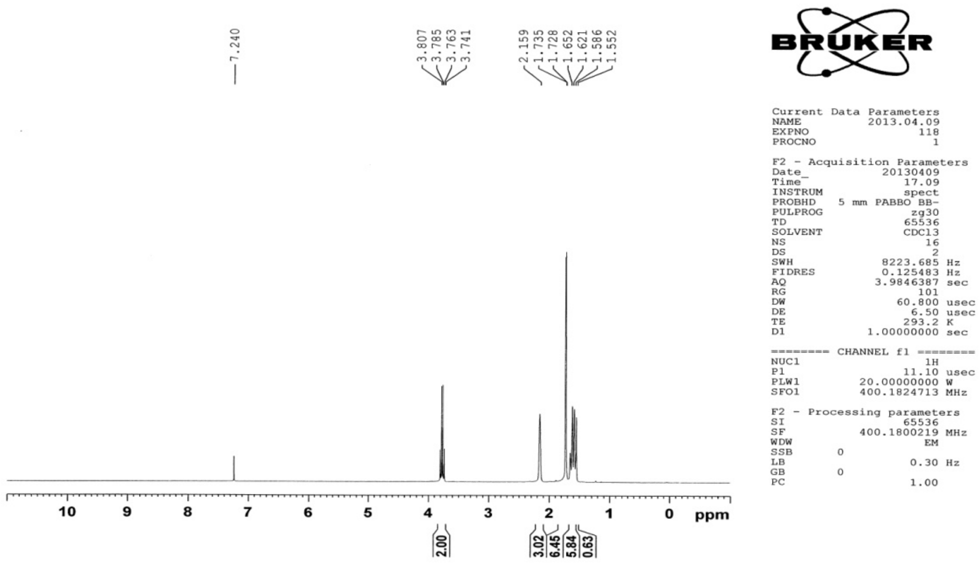

Figure S17. ^{1}H NMR spectrum of 1-adamantyl 2,2,2-trifluoroethyl ether.

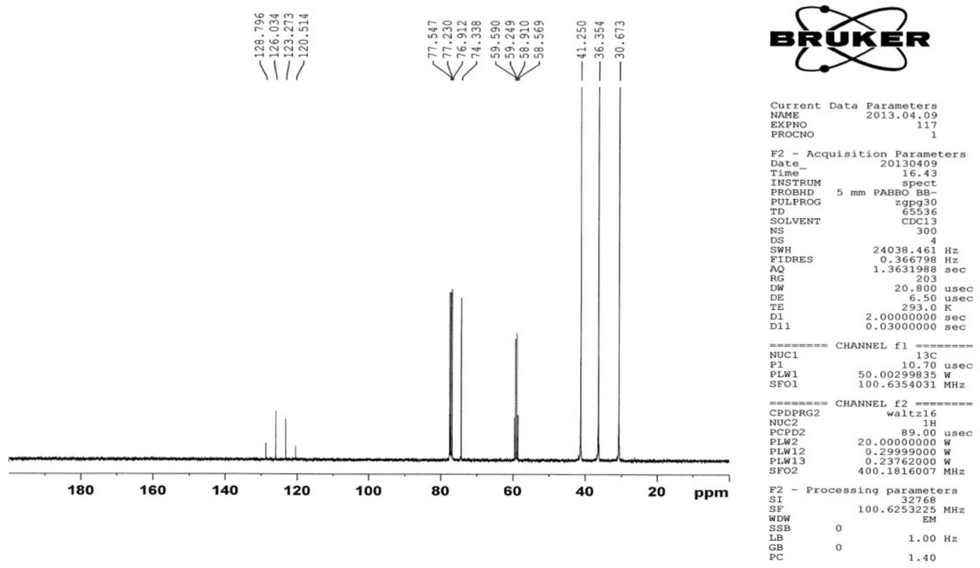

Figure S18. ^{13}C NMR spectrum of 1-adamantyl 2,2,2-trifluoroethyl ether.

**Substrate:** 1-adamantyl 2,2,2-trifluoroethyl ether (5.588×10^{-3}M)	**Gas chromatography :** GC-9A (Shimadzu)
**Solvent:** *n*-Pentane	**Column :** glass (length: 2.1m, O.D: 5mm, I.D: 3mm)
**Injection Temp. :** 210 °C	**Stationary phase :** Carbowax 20M 10% Chromosorb WAW 80/100
**Column Temp. :** 170 °C	
**Injection volume :** 1 μL	**Detector :** FID, **Carrier gas:** N_2

START

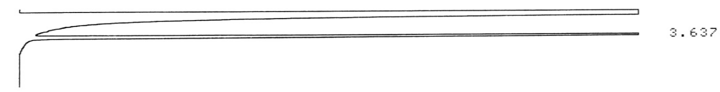

CHROMATOPAC C-R3A FILE 0
SAMPLE NO 0 METHOD 41
REPORT NO 697

PKNO	TIME	AREA	MK	IDNO	CONC	NAME
1	3.637	125972			100	
	TOTAL	125972			100	

Figure S19. G.C. analysis sheet of 1-adamantyl 2,2,2-trifluoroethyl ether.

1-ADSCOCL IN 80ETOH (A)
ERROR 1:INVALID SYNTAX
START

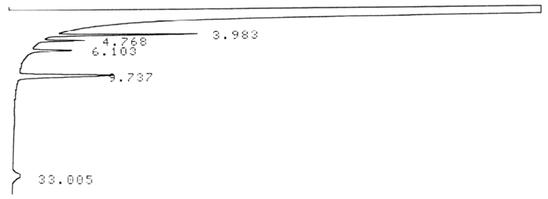

CHROMATOPAC C-R3A FILE 0
SAMPLE NO 0 METHOD 41
REPORT NO 640

PKNO	TIME	AREA	MK	IDNO	CONC	NAME
1	3.983	5738			27.1727	
2	4.768	1975			9.3527	
3	6.103	2635			12.4789	
4	9.737	8768			41.5217	
5	33.005	2001			9.474	
	TOTAL	21117			100	

1-ADSCOCL IN 80ETOH (D)
ERROR 1:INVALID SYNTAX
START

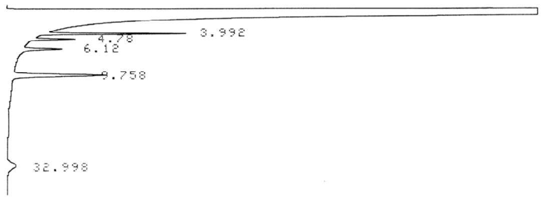

HROMATOPAC C-R3A FILE 0
AMPLE NO 0 METHOD 41
EPORT NO 641

KNO	TIME	AREA	MK	IDNO	CONC	NAME
1	3.992	5557			27.3626	
2	4.78	1903			9.3706	
3	6.12	2502			12.3182	
4	9.758	8428			41.5012	
5	32.998	1919			9.4475	
	TOTAL	20309			100	

Figure S20. G.C. analysis sheets of products of 1-adamantyl chlorothioformate solvolysis in 80% EtOH.

1-ADSCOCL IN 60ACT (A)
ERROR 1:INVALID SYNTAX
START

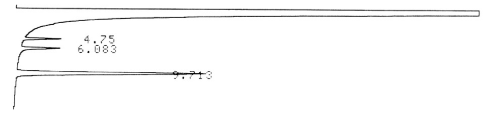

CHROMATOPAC C-R3A FILE 0
SAMPLE NO 0 METHOD 41
REPORT NO 630

PKNO	TIME	AREA	MK	IDNO	CONC	NAME
1	4.75	1914			7.7849	
2	6.083	2821			11.4759	
3	9.713	19848			80.7391	
	TOTAL	24583			100	

STOP
1-ADSCOCL IN 60ACT (B)
ERROR 1:INVALID SYNTAX
START

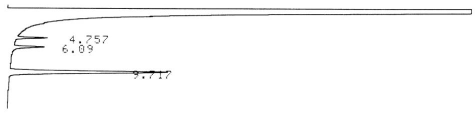

CHROMATOPAC C-R3A FILE 0
SAMPLE NO 0 METHOD 41
REPORT NO 631

PKNO	TIME	AREA	MK	IDNO	CONC	NAME
1	4.757	1538			7.5694	
2	6.09	2209			10.8741	
3	9.717	16569			81.5564	
	TOTAL	20316			100	

Figure S21. G.C. analysis sheets of products of 1-adamantyl chlorothioformate solvolysis in 60% Me_2CO.

1-ADSCOCL 70TFE)(A)
ERROR 1:INVALID SYNTAX
START

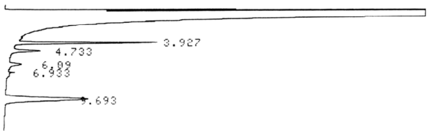

CHROMATOPAC C-R3A FILE 0
SAMPLE NO 0 METHOD 41
REPORT NO 674

PKNO	TIME	AREA	MK	IDNO	CONC	NAME
1	3.927	5160			34.016	
2	4.733	1818			11.9838	
3	6.09	901			5.9418	
4	9.693	7289			48.0584	
	TOTAL	15168			100	

1-ADSCOCL IN 70TFE (B)
ERROR 1:INVALID SYNTAX
START

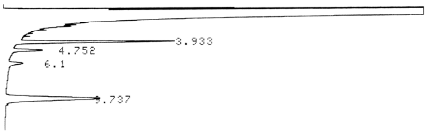

CHROMATOPAC C-R3A FILE 0
SAMPLE NO 0 METHOD 41
REPORT NO 671

PKNO	TIME	AREA	MK	IDNO	CONC	NAME
1	3.933	5968			37.6379	
2	4.752	1757			11.083	
3	6.1	1229			7.7505	
4	9.737	6902			43.5285	
	TOTAL	15857			100	

Figure S22. G.C. analysis sheets of products of 1-adamantyl chlorothioformate solvolysis in 70% TFE.

START

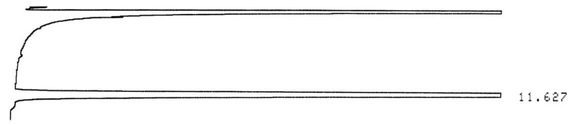

CHROMATOPAC C-R3A FILE 0
SAMPLE NO 0 METHOD 41
REPORT NO 949

PKNO	TIME	AREA	MK	IDNO	CONC	NAME
1	11.627	134277			100	
	TOTAL	134277			100	

START

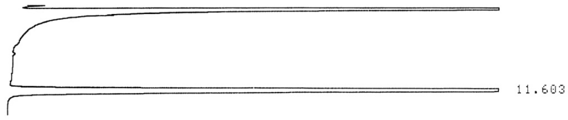

CHROMATOPAC C-R3A FILE 0
SAMPLE NO 0 METHOD 41
REPORT NO 950

PKNO	TIME	AREA	MK	IDNO	CONC	NAME
1	11.603	102519			100	
	TOTAL	102519			100	

Figure S23. G.C. analysis sheets of products of 1-adamantyl fluorothioformate solvolysis in 100% EtOH.

63E-A
ERROR 1:INVALID SYNTAX
START

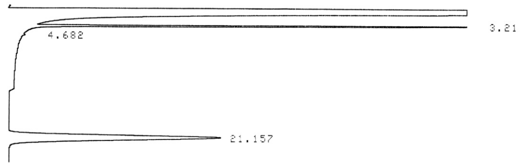

CHROMATOPAC C-R3A FILE 0
SAMPLE NO 0 METHOD 41
REPORT NO 742

PKNO	TIME	AREA	MK	IDNO	CONC	NAME
1	3.21	36932			40.4901	
2	21.157	54280			59.5099	
	TOTAL	91212			100	

63E-A
ERROR 1:INVALID SYNTAX
START

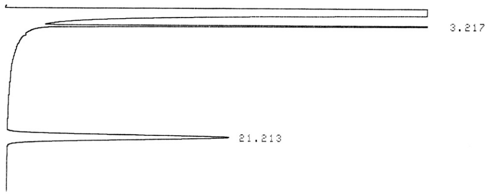

CHROMATOPAC C-R3A FILE 0
SAMPLE NO 0 METHOD 41
REPORT NO 733

PKNO	TIME	AREA	MK	IDNO	CONC	NAME
1	3.217	38027			40.2068	
2	21.213	56552			59.7932	
	TOTAL	94579			100	

Figure S24. G.C. analysis sheets of products of 1-adamantyl fluorothioformate solvolysis in 60% EtOH.

70T-A
ERROR 1:INVALID SYNTAX
START

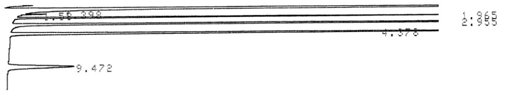

CHROMATOPAC C-R3A FILE 0
SAMPLE NO 0 METHOD 41
REPORT NO 786

PKNO	TIME	AREA	MK	IDNO	CONC	NAME
1	1.398	446			0.3641	
2	1.965	20263			16.5549	
3	2.955	62183			50.8027	
4	4.378	31337			25.6018	
5	9.472	8172			6.6765	
	TOTAL	122401			100	

70T-B
ERROR 1:INVALID SYNTAX
START

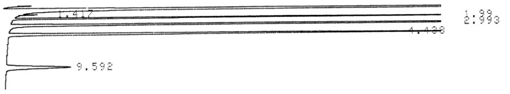

CHROMATOPAC C-R3A FILE 0
SAMPLE NO 0 METHOD 41
REPORT NO 787

PKNO	TIME	AREA	MK	IDNO	CONC	NAME
1	1.417	324			0.2755	
2	1.99	19561			16.6309	
3	2.993	59409			50.5093	
4	4.438	30281			25.7448	
5	9.592	8044			6.8394	
	TOTAL	117620			100	

Figure S25. G.C. analysis sheets of products of 1-adamantyl fluorothioformate solvolysis in 70% TFE.

60T40E (A)
ERROR 1:INVALID SYNTAX

START

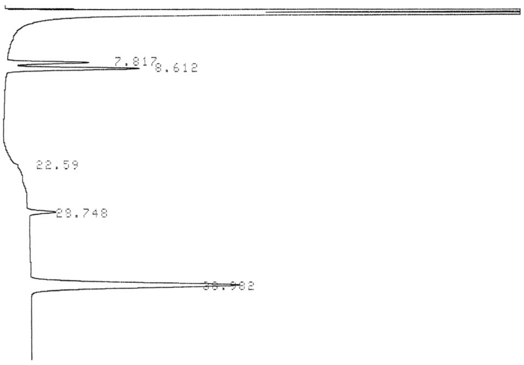

CHROMATOPAC C-R3A FILE 0
SAMPLE NO 0 METHOD 41
REPORT NO 1206

PKNO	TIME	AREA	MK	IDNO	CONC	NAME
1	7.817	14827			10.903	
2	8.612	26988	V		19.845	
3	22.59	253			0.1858	
4	28.748	5892			4.3327	
5	38.982	88035			64.7337	
	TOTAL	135995			100	

Figure S26. G.C. analysis sheet of products of 1-adamantyl fluorothioformate solvolysis in 60T-40E.
